# Experiences and Challenges with Congenital Hypothyroidism Newborn Screening in Indonesia: A National Cross-Sectional Survey

**DOI:** 10.3390/ijns10010008

**Published:** 2024-01-19

**Authors:** Aman Bhakti Pulungan, Helena Arnetta Puteri, Muhammad Faizi, Paul Leslie Hofman, Agustini Utari, Jean-Pierre Chanoine

**Affiliations:** 1 Department of Child Health, Faculty of Medicine, Universitas Indonesia, Jakarta 10430, Indonesia; 2 Global Pediatric Endocrinology and Diabetes, Vancouver, BC V5Z 4R3, Canada; agustiniutari@gmail.com (A.U.); jchanoine@cw.bc.ca (J.-P.C.); 3 Indonesian Pediatric Society, Jakarta 10430, Indonesia; muhammad.faizi@fk.unair.ac.id; 4 Global Child Health Foundation, Jakarta 10430, Indonesia; helenaarnetta@gmail.com; 5 Faculty of Medicine, Universitas Indonesia, Jakarta 10430, Indonesia; 6 Department of Child Health, Faculty of Medicine, Universitas Airlangga—Dr. Soetomo General Hospital, Surabaya 60286, Indonesia; 7 Liggins Institute, University of Auckland, Auckland 1023, New Zealand; p.hofman@auckland.ac.nz; 8 Department of Child Health, Faculty of Medicine, Universitas Diponegoro, Semarang 50275, Indonesia; 9 Endocrinology and Diabetes Unit, British Columbia Children’s Hospital, Vancouver, BC V6H 3N1, Canada

**Keywords:** newborn screening, congenital hypothyroidism, experiences, challenges

## Abstract

The expansion of newborn screening (NBS) for congenital hypothyroidism (CH) is essential to reducing the number of preventable intellectual disabilities in children. Because of logistical issues, including geographic extremes, distinct cultures, and 4.8 million births annually, Indonesia has struggled to achieve universal NBS coverage. A national cross-sectional electronic survey was conducted to explore challenges in CH NBS. Responses from 423 healthcare professionals and program administrators across 30 provinces in Indonesia were collected. The major challenges reported were refusal from families (39.2%), newborns being discharged <24 h (38.3%), and limited availability of filter paper (35.9%). The respondents considered refusal from families to be due to fear, while others did not understand the necessity of CH NBS. The vast majority of respondents believed that parents do not have sufficient understanding regarding CH NBS (96.5%). Our study found that only 38.5% of respondents had received formal CH NBS training, with pediatric endocrinologists being the only profession in which all respondents had been trained. Concerted efforts are needed to improve the access to and availability of resources, increase the capacity for sample collection and analysis, empower healthcare professionals, and develop educational resources to promote understanding and acceptance of NBS amongst families.

## 1. Introduction

With an estimated global incidence ranging from 1:2000 to 1:4000 live births, congenital hypothyroidism (CH) is the most common preventable cause of intellectual disability [1,2,3,4]. Early identification and treatment of affected newborns through national, whole-population screening has been shown to be effective in preventing developmental sequelae. Newborn screening (NBS) has been hailed as one of the greatest public health successes globally. CH NBS was first developed in 1972 in Quebec, Canada, by Professor Jean Dussault [5]. Since then, most high-income countries have successfully implemented NBS programs. However, the same cannot be said for a large number of low- and middle-income countries (LMICs), where the majority of newborns are not screened for CH [6,7]. The introduction and continuing expansion of NBS across the world is a critical step to achieving the Sustainable Development Goals Nos. 3.2 and 3.4 to reduce the premature morbidity and mortality of neonates due to non-communicable diseases [8].

Indonesia is the world’s largest archipelago, the fourth-most populated country, and has the fifth-most births internationally, with 4.8 million births annually [9]. In May 1999, eleven Southeast Asian countries, including Indonesia, convened for the Workshop on National Neonatal Screening for CH, where a consensus was made to initiate and develop regional CH NBS programs [10]. With the support of the International Atomic Energy Agency (IAEA), Indonesia began a pilot study involving Dr. Cipto Mangunkusumo Hospital in Jakarta and Dr. Hasan Sadikin Hospital in Bandung from 2000 to 2005 [11,12]. Upon the completion of a health technology assessment (HTA) in 2006, the CH NBS program expanded to eight provinces in 2008 (West Sumatra, Jakarta, West Java, Central Java, East Java, Yogyakarta, Bali, and South Sulawesi), and in 2014, the Ministry of Health (MOH) released a decree mandating CH NBS [13]. While CH NBS has subsequently continued to expand to other provinces, national CH NBS coverage has remained low and was reported to be 2.3% in 2022 [14]. This prompted the MOH to relaunch CH NBS in August 2022 [15], followed by the release of a national policy that makes CH NBS a requirement for all healthcare professionals to claim delivery payments from the national insurance in September 2023 [16].

Although most high-income countries have achieved universal screening coverage, Indonesia is still working towards developing a cost-effective, efficient approach as well as increasing awareness among the general public and healthcare professionals regarding NBS. The aim of this study was to identify barriers preventing the wider uptake of NBS in Indonesia. To achieve this, a national, cross-sectional online survey was conducted by Global Pediatric Endocrinology and Diabetes (GPED, www.globalpedendo.org [accessed on 2 December 2023]) and the Endocrinology Working Group of the Indonesian Pediatric Society (IPS). The focus was on identifying and exploring the experiences of healthcare professionals and program administrators in CH NBS. This is the first national survey to be conducted on CH NBS in Indonesia with respondents directly involved in the national program’s implementation.

## 2. Materials and Methods

The survey was conducted in conjunction with a national webinar hosted by GPED, a non-profit organization with a focus on improving the care of children with endocrine disorders in LMICs, and the Endocrinology Working Group of IPS. All health professionals self-registering as interested by neonatal congenital hypothyroidism screening in Indonesia could attend this free webinar. However, the organizers actively reached out to members of IPS, the Indonesian Midwives Association and MOH public health office networks for the promotion of this webinar. Survey questions were disseminated at the end of the webinar on 3 September 2023. Healthcare professionals (n = 957) joined via Zoom and YouTube Live. Webinar participants who consented to the survey provided responses. Google Forms, a web-based survey software, was used to collect and store responses in a digital spreadsheet.

The survey consisted of four main sections, beginning with an informed consent section that detailed the objectives of the survey. After consent was received, respondents were directed to the next section, which comprised five questions on their demographic background. The final question in this section screened for webinar participants who worked in health facilities that provide CH NBS. Those who confirmed that they worked in health facilities that conduct CH NBS were eligible to proceed with the survey questions described in the third section. This third section consisted of seven questions on the challenges of CH NBS experienced by each respondent in their respective health facilities. The fourth and final section of the survey explored the attitudes of respondents towards CH NBS through eight “yes” or “no” questions. The survey took approximately 5 to 10 min to complete.

The subjects included in this study were healthcare professionals and administrative staff of public health offices working in health facilities directly involved in the CH NBS program across Indonesia. During the data analysis process, duplicate responses were removed and descriptive statistics were used. Responses to open-ended questions were summarized by approximating the similarities in semantic content. The answers to the survey questions were treated anonymously. Ethics approval was not required for this survey.

## 3. Results

### 3.1. Respondent Demographics

A total of 481 responses were gathered in the survey disseminated during the GPED-IPS joint webinar. After removing duplicate responses, a total of 423 responses were included in the final analysis. Consistent with a national webinar that included hundreds of participants, there was great diversity in terms of the demographic backgrounds of the respondents. The demographic profiles of the respondents are shown in Table 1.

There were over a dozen different professions involved in our study, with the majority of participants being split between midwives (28.8%), general practitioners (22.9%), and pediatricians (21.5%). Besides clinicians involved in NBS, our survey also attracted professions involved in the sample analysis and public health aspect of NBS. Our study included representatives from each health facility level in Indonesia, with most participants working at type C hospitals (30.3%) and primary health centers (20.6%). Finally, out of 38 provinces in Indonesia, there were representatives from 30. Most respondents were from the Java region, notably East Java, West Java, and Central Java (23.4%, 13.2%, and 11.8%, respectively). Other responses were spread across other provinces located on the major islands, such as Kalimantan, Sumatera, Sulawesi, and Papua. 

### 3.2. Implementation of Newborn Screening for Congenital Hypothyroidism

Table 2 summarizes the current state of CH NBS implementation among the facilities represented by the respondents. Most respondents reported that NBS for CH had been initiated in their respective health facilities over the last 3 years. One third of respondents indicated that their healthcare facility has not been able to screen all newborns, primarily because of insufficient availability of filter paper (33.1%), refusal from families (19.8%), or the NBS not being viewed as a compulsory national program before September 2023 (15.4%). 

### 3.3. Challenges in Congenital Hypothyroidism Newborn Screening

#### 3.3.1. General Challenges in National Program Implementation

The challenges uncovered in our study are summarized in Table 3. The three major challenges reported by respondents were refusal from families (39.2%), sampling time difficulty as newborns were discharged at <24 h of age (38.3%), and limited availability of filter paper (35.9%). In addition to NBS, our study explored whether levothyroxine was available for CH management. There were a concerning large number of responses indicating that levothyroxine was not available (31.2%), while 43.3% reported that it was consistently available.

#### 3.3.2. Challenges from the Parents’ Perspectives

Our study explored the reasons behind parent’s refusal through the perspectives of healthcare professionals (Table 4). Most respondents reported that one of the reasons for parental refusal is the fear of seeing their newborn having blood drawn from their heel (49.4%), while others do not understand the necessity of CH NBS (31%). There are also general fears regarding the CH NBS procedure and potential side effects (32.2%). There were several respondents who reported never encountering parents who refused screening (15.6%).

Our survey also explored the healthcare professionals’ experience with parents’ education levels in the field (Table 5). Almost all respondents shared their belief the parents do not have sufficient knowledge and understanding regarding CH NBS (96.5%). When asked regarding platforms to educate parents about CH NBS, most participants agreed that this should be incorporated into routine antenatal care (53.2%). Others supported the idea of educational content through social media (20.8%) and community health center outreach activities (17.3%).

#### 3.3.3. Challenges from the Perspectives of Healthcare Professionals

Challenges highlighted by healthcare professionals conducting NBS mainly revolved around a lack of training and technical skills in sample collection. Our study found that only 38.5% of respondents had received formal CH NBS training (Table 6). Only 23.7% to 66.7% of the respondents within each group of profession had received training regarding CH NBS. Pediatric endocrinologists were the only profession reported in our study where all respondents had been trained specifically regarding CH NBS. This result was independent from the type of healthcare facility. For public healthcare facilities, 147 respondents (37.7%) stated that they have received CH NBS training while 243 respondents (62.3%) have not. For private healthcare facilities, 16 respondents stated they have received training (48.5%), while 17 (51.5%) have not (chi-square test 1.50, *p* = 0.22). The large majority of respondents echoed the need for additional training regarding NBS (93.6%).

### 3.4. Attitudes towards Congenital Hypothyroidism Newborn Screening

The final section of our survey explored the attitudes of healthcare professionals and staff involved in CH NBS towards CH NBS itself (Table 7). The vast majority of respondents had positive attitudes towards CH NBS. While most respondents felt confident enough to educate and answer parents’ questions regarding NBS (87%), fewer felt that they had sufficient understanding regarding the CH NBS system in Indonesia (69.5%). 

## 4. Discussion

With 423 responses from ten different professional backgrounds spread across 30 provinces, the findings of this study highlight the specific challenges still facing CH NBS implementation in Indonesia. Despite being initiated over two decades ago, the national coverage of CH NBS is still low, with data from 2022 revealing a coverage of only 2.3% of all newborns. Encouragingly, the country has seen a significant increase in government commitment and support for the CH NBS national program. Unpublished data from the MOH show that the number of samples increased steadily in 2023, standing at 48,887 samples a week in the third week of October 2023. This brings the national report of CH NBS samples collected in 2023 to 692,744 as of the third week of October 2023, translating to a national coverage of 15.53% [16]. However, current reports show that there is still great disparity between provinces, with reported coverage from as low as 0.02% in South Papua to 40.54% in Bali [16].

CH NBS in Indonesia has progressed from being the subject of a pilot study involving two hospitals to being implemented across 38 provinces. Without laboratories specifically designated for newborn screening, our CH NBS program relies on referral laboratories appointed by the MOH. Public health centers, hospitals, and other healthcare facilities that conduct NBS send samples to their appointed referral laboratory for the initial screening of CH. Positive results are then reported back to the healthcare facility of origin to arrange further tests as needed. While several referral laboratories have been assigned since 2008, our study reveals that the majority of healthcare facilities only started screening for CH in 2023 (40%), with many reporting that the recent MOH policy making CH NBS compulsory to claim newborn delivery payments from the national insurance acted as the catalyst for CH NBS initiation. Very few respondents (1.4%) reported that the implementation began within the first decade the program was introduced in Indonesia.

The implementation of a national NBS program is complex, with the involvement of multiple stakeholders, and comprises several areas, including education, screening, follow-up of results, diagnosis, management, and evaluation, all of which must be institutionalized within national public health systems. In developing countries, especially in the Southeast Asian and North African regions, the initiation and implementation of NBS programs are limited by a number of challenges from varying public health priorities, such as low socio-economic status and poor parental awareness [10,17,18]. Our study found that Indonesia is also still limited by these challenges.

One of the most common problems mentioned by respondents was difficulty with sampling time, as patients tended to be discharged less than 24 h after birth. Studies conducted in Sri Lanka and Malaysia have also found this to be a common hurdle [19,20]. The American Academy of Pediatrics recommends that the optimal time for testing is between 48 and 72 h of age [21]. With the initial TSH surge post-birth, specimens collected within the first 24 h are associated with higher false positive rates [22]. From a cost–benefit perspective, high false positive rates are unfavorable due to negative psychological effects and stress on families, as well as the increased costs associated with confirmation tests [23,24]. In Indonesia, while the costs of the initial CH NBS are covered by the MOH, currently, not all confirmation tests for patients are covered. Patients must either arrange for further testing in the referral laboratories located in major cities or pay out-of-pocket with other providers. A solution often suggested by experts is to introduce higher cutoffs for specimens collected <24 h of age. However, it must be noted that introducing higher cutoffs comes with the risk of missing a true case of CH [25].

In addition to early discharge of patients, Indonesia faces added challenges such as the fact that not all births take place in healthcare facilities and not all families have general care physicians that provide long-term care. Most antenatal and postpartum care of mothers and children in the country is provided by midwives. A study by Efendi et al. found that only 55.2% of Indonesian mothers seek birth assistance from healthcare providers [26]. Sri Lanka is another Southeast Asian country that started CH NBS programs within the last decade, and almost 99% coverage was achieved within four months of its implementation [20]. Such success was possible as the vast majority of births took place in hospitals or in maternity homes. Similarly, high-income countries, where births generally take place in maternity units, have also reported coverage exceeding 99.5% [27]. Learning from the experiences of other countries where universal coverage for screening has been achieved, Indonesia needs to consider revising the current regulations and guidelines for CH NBS or making adjustments to national health insurance policies for neonatal and postpartum care. While changes are needed to improve NBS uptake, the major logistical issues associated with such a large newborn population and the distribution of births over many islands needs emphasizing. Indonesia has unique challenges, and these will take time to resolve.

Building a sustainable CH NBS program depends on the availability of robust infrastructure equipped with adequate logistical support. The findings of this study reveal that another major challenge in achieving universal screening coverage is the lack of basic consumables, such as the availability of NBS papers. At the time this study was conducted, Indonesia only had twelve designated national referral laboratories, limiting the number of samples that can be analyzed each week and further complicating the process with lengthy processing and reporting times. The current national capacity for screening is still below the recommendations of the Working Group of Neonatal Screening of the European Society for Pediatric Endocrinology, which states that screening should be conducted in centralized laboratories that cover 100,000 newborns per year [28]. Even with the increasing government commitment and support, universal screening coverage cannot be achieved without adequate facilities and infrastructural support.

Parental understanding, perspectives, and concerns surrounding CH NBS is a critical factor that determines the success of a national NBS program. Parents need to be well-informed regarding CH and the benefits of NBS to provide the necessary consent, and this remains a challenge in Indonesia. A study by Biswas et al. on parental perception towards newborn metabolic screening in Indonesia found that, once parents understood the principles behind metabolic testing, they consented to screening [29]. While most respondents felt confident about educating parents (87%), they found that Indonesian parents do not have sufficient knowledge and understanding regarding CH NBS (96.5%). The American College of Obstetrics and Gynecology recommends that NBS information should be included during prenatal visits through the postpartum period, with the optimal time to provide NBS education being the third trimester of pregnancy [30]. As CH is the first disorder to be identified for a national NBS program in Indonesia, the public is largely unaware of the purpose and benefits of NBS. A study by Rama Devi et al. shared the experience of initiating an NBS program in a rural area of Andhra Pradesh, India, and reported that, with repeated awareness and training regarding NBS, the program was able to be a success despite initial struggles with the local population’s illiteracy, ignorance, and taboos, as well as a lack of awareness and interest amongst the medical community [31]. Parental education regarding the importance and relevance of NBS should be incorporated across maternal and childcare services, including campaigns targeted to increase awareness of NBS, inclusion in the national mother and child health book, and a national consensus that ensures expecting parents are educated regarding NBS regardless of the individual providing their antenatal care.

Our survey was conducted alongside a national webinar hosted by GPED and the Endocrinology Working Group of IPS. Even though this study does not directly assess the level of knowledge of healthcare professionals in Indonesia, the questions submitted by our webinar participants were mainly basic questions surrounding CH as a disorder and the CH NBS concept, process, and benefits. We can see that healthcare professionals in Indonesia still lack the knowledge and understanding needed to educate parents to instill trust and elicit acceptance of CH NBS. Our study also found that pediatric endocrinologists are the only profession to receive formal CH NBS training. This prompted most respondents to express the need for additional training regarding NBS (93.6%). These training programs should include hands-on workshops targeted at healthcare professionals directly involved in sample collection, which, in our case, are mainly midwives. Finally, a socialization program regarding the CH NBS system also needs to be established, as only 69.5% of respondents felt that they had sufficient understanding regarding the CH NBS system in Indonesia.

While the initial focus of the NBS program will be on increasing screening coverage in Indonesia, the program’s success also involves contacting and appropriately treating affected infants. Patients with positive tests must undergo confirmation tests, and ultimately, once diagnosed with CH, access to and availability of treatment must be ensured. While levothyroxine is widely available, as of 2023, there are only 39 pediatric endocrinologists nationally [32]. Therefore, currently, pediatric endocrinologists work with pediatricians to manage CH. As we gradually work towards universal screening for CH and anticipate more confirmed CH cases, adequate training and distribution of pediatricians capable of managing CH need to be established nationally.

As Indonesia continues to work towards improving national coverage for CH NBS and developing NBS for other disorders, such as congenital adrenal hyperplasia and G6PD, we hope that the findings of this study can provide preliminary data to help shape and refine existing policies and programs to reach the ultimate goal of ensuring that every newborn in Indonesia is screened.

## 5. Limitations

Although we included representatives of over ten different professional backgrounds, additional perspectives are required, including those from parents and obstetricians. This study mainly reflects the experiences of healthcare professionals and staff involved in CH NBS in Indonesia, but does not objectively assess respondents’ knowledge and attitudes. The vast majority of the respondents were from public healthcare facilities and may also play more than one role, for instance, both clinical and administrative. There was also a lack of representation from healthcare professionals working in private healthcare facilities. As CH screening improves, future surveys should take into account this limitation, provide the opportunity to list more than one role in the process of CH screening, and actively reach out to those working in private healthcare facilities. Lastly, further studies aiming to gather quantitative data regarding CH NBS in Indonesia should recruit more respondents to provide better national representation. 

## 6. Conclusions

CH NBS in Indonesia faces challenges such as a lack of logistical and infrastructural support, lack of knowledge and training of healthcare professionals, and hesitation from families. With the complexity of developing and maintaining a national program of this scale, funding is not the only driver of success. Our study highlights that concerted efforts must be made to improve the access to and availability of resources, increase the capacity for sample collection and analysis, educate and empower healthcare professionals, and develop educational materials and programs to promote the understanding and acceptance of NBS amongst families.

## Figures and Tables

**Table 1:** Respondent demographics.

Respondent Demographics	n (%)	
Profession (n = 423)		
Midwives	122 (28.8)	
General practitioners	97 (22.9)	
Pediatricians	91 (21.5)	
Pediatric endocrinologists	11 (2.6)	
Clinical pathologists	25 (5.9)	
Medical laboratory technologists	18 (4.3)	
Nurses	37 (8.8)	
Public health officers	5 (1.2)	
Screening program administrators	6 (1.4)	
Others	11 (2.6)	
Health facility level (n = 423)		
Public health office	8 (1.9)	
Referral laboratory	1 (0.2)	
Primary health center	87 (20.6)	
Type A hospital *	39 (9.2)	
Type B hospital *	76 (18.0)	
Type C hospital *	128 (30.3)	
Type D hospital *	51 (12.1)	
Private clinics/hospitals	33 (7.8)	

* Type A hospitals are national referral hospitals that cater to between 13 and 20 subspecialties. Type B hospitals provide the services of 4 basic specialties, 4 medical support specialties, 8 other specialties, and 2 basic subspecialties. Type C hospitals have 4 basic specialties and 4 medical support specialties. Type D hospitals have 2 basic specialties.

**Table 2:** Implementation of congenital hypothyroidism newborn screening.

Responses	n (%)
Since when has your health facility implemented the CH NBS policy? (n = 423)	
2000–2010	6 (1.4)
2011–2020	61 (14.4)
2021–2022	55 (13.0)
2023	169 (40.0)
Unsure/unaware	132 (31.2)
Are all babies born in your health facility screened? (n = 423)	
Yes	287 (67.8)
No	136 (32.2)
If you answered no to the previous question, provide your reason… (n = 136)	
Limited stock of filter paper	45 (33.1)
Refusal from families	27 (19.8)
NBS was not compulsory previously	21 (15.4)
Healthy infants discharged <24 h post birth	14 (10.3)
Critically ill newborns	10 (7.4)
Lack of trained healthcare professionals for sample collection	6 (4.4)
Only patients with ID cards in the respective cities can be screened	5 (3.7)
Infants without national health insurance	4 (2.9)
Sample delivery problems	1 (0.7)
No reason provided	3 (2.2)

**Table 3:** Challenges in CH NBS implementation.

Responses	n (%)
Have you experienced any of the following difficulties in implementing CH NBS? *	
Parents who refuse screening	166 (39.2)
Sampling time is difficult because infants are often discharged <24 h	162 (38.3)
Limited availability of filter paper	152 (35.9)
Technical difficulties in sample collection	107 (25.3)
Difficulties in sending samples to referral labs	73 (17.3)
Difficulty with re-screening/confirmation tests on positive screening results	49 (11.6)
Financial barriers to confirmation tests	63 (14.9)
Others:	
Results of screening that take a long time	5 (1.2)
Lack of trained healthcare professionals for sample collection	5 (1.2)
Financial challenges (non-NHI patients, OOP when filter papers are unavailable) **	4 (1.0)
Geographical barriers	2 (0.5)
Confusion on whether to screen in special cases (sick infants, premature infants, etc.)	1 (0.2)
Lack of collaboration with referral labs	1 (0.2)
Patients who do not come to the health facility for their scheduled screening	1 (0.2)
Have not experienced difficulty	25 (5.9)
Is levothyroxine available for CH management in your health facility? (n = 423)	
Yes	183 (43.3)
No	132 (31.2)
Unaware/unsure	108 (25.5)

* Respondents were allowed to select multiple responses. ** NHI: national health insurance, OOP: out-of-pocket.

**Table 4:** Refusal of CH NBS screening by Indonesian parents.

Responses	n (%)
If you have ever encountered parents who refused screening, what were their reasons for refusing to be screened? *	
Can’t bear to see their child having blood drawn on the heel	209 (49.4)
Afraid	136 (32.2)
Don’t feel the need	131 (31.0)
Hearing fake news related to NBS	45 (10.6)
Religious beliefs	43 (10.2)
Additional financial expenditure (non-NHI patients, filter papers unavailable) **	5 (1.2)
Healthcare professionals who were unable to provide adequate and clear explanations regarding NBS and its benefits	2 (0.5)
Have never met parents who refused screening	66 (15.6)

* Respondents were allowed to select multiple responses. ** NHI: national health insurance.

**Table 5:** Indonesian healthcare professionals’ perspectives towards parental understanding.

Responses	n (%)
Do you think parents in Indonesia have sufficient knowledge regarding CH NBS? (n = 423)	
Yes	15 (3.5)
No	408 (96.5)
If you answered “No” to the previous question, what form of education do you think is effective for parents in Indonesia? *	
Education during routine antenatal care	225 (53.2)
Educational content through social media	88 (20.8)
Community health center outreach activities	73 (17.3)
Educational material in a book/booklet	17 (4.0)
Door to door education	10 (2.4)
Online seminars	6 (1.4)
Offline seminars	3 (0.7)
Revise the national maternal and child health book	1 (0.2)

* Respondents were allowed to select multiple responses.

**Table 6:** Training of healthcare professionals on congenital hypothyroidism newborn screening.

Responses	n (%)
Have you ever received formal training regarding CH NBS? (n = 423)	
Yes	163 (38.5)
No	260 (61.5)
Respondents who have received training based on healthcare profession	
Midwives	42/122 (34.4)
General practitioners	23/97 (23.7)
Pediatricians	51/91 (56.0)
Pediatric endocrinologists	11/11 (100)
Nurses	11/37 (29.7)
Clinical pathologists	12/25 (48.0)
Medical laboratory technologists	5/18 (27.8)
Public health officers	2/5 (40.0)
Screening program administrators	4/6 (66.7)
Do you feel you need additional training regarding CH NBS? (n = 423)	
Yes	396 (93.6)
No	27 (6.4)
If yes, what form should this training take place? *	
Offline seminars	169 (42.7)
Online seminars	130 (32.8)
Educational material in a book/booklet	87 (22.0)
Educational content through social media	17 (4.3)
Hands on workshop	14 (3.5)

* Respondents were allowed to select multiple responses.

**Table 7:** Attitudes towards congenital hypothyroidism newborn screening.

Responses	n (%)
Does CH NBS add additional burden to your work? (n = 423)	
Yes	55 (13.0)
No	368 (87.0)
Does CH NBS provide benefits in improving children’s health? (n = 423)	
Yes	421 (99.5)
No	2 (0.5)
Have you received sufficient training related to CH NBS? (n = 423)	
Yes	197 (46.6)
No	226 (53.4)
Do you feel confident enough to educate and answer parents’ questions regarding CH NBS? (n = 423)	
Yes	368 (87.0)
No	55 (13.0)
Do you feel like you have sufficient understanding about the CH NBS in Indonesia, including the referral system, algorithm of positive results, etc.? (n = 423)	
Yes	294 (69.5)
No	129 (30.5)
Should all newborns be screened? (n = 423)	
Yes	406 (96.0)
No	17 (4.0)
Is newborn screening the responsibility of all health workers? (n = 423)	
Yes	399 (94.3)
No	24 (5.7)
Should Indonesia conduct screening for other congenital diseases such as congenital adrenal hyperplasia (CAH)? (n = 423)	
Yes	398 (94.1)
No	25 (5.9)

## Data Availability

Data are contained within the article.
